# The synthesis of alternating donor–acceptor polymers based on pyrene-4,5,9,10-tetraone and thiophene derivatives, their composites with carbon, and their lithium storage performances as anode materials†

**DOI:** 10.1039/d1ra00794g

**Published:** 2021-04-22

**Authors:** Xin Guo, Qing Yuan, Chunxia Li, Hongmei Du, Jinsheng Zhao, Lixia Liu, Yunwu Li, Yu Xie, Vijay Vaidya

**Affiliations:** Shandong Key Laboratory of Chemical Energy Storage and Novel Cell Technology, Liaocheng University Liaocheng 252059 P. R. China j.s.zhao@163.com liyunwu@lcu.edu.cn; College of Chemistry and Chemical Engineering, Liaocheng University 252059 P. R. China; College of Environment and Chemical Engineering, Nanchang Hangkong University Nanchang 330063 PR China xieyu_121@163.com; Fu Technology, Co. Ltd Tianjin 851000 P. R. China

## Abstract

Two conjugated polymer@activated carbon composites were synthesized by the *in situ* polymerization of two donor–acceptor type polymers including poly[(thiophene-2,5-yl)-((pyrene-4,5,9,10-tetraone)-2,7-yl)] (PTPT) and poly[((2,3-dihydrothieno[3,4-b][1,4]dioxine)-5,7-yl)-((pyrene-4,5,9,10-tetraone)-2,7-yl)] (POTPT) on activated carbon (AC) by one-step cross-coupling reaction catalyzed by an organometallic catalyst. Cyclic voltammetry showed that both polymers exhibited ambipolar properties, low bandgaps, and low electrode potentials, which could be useful for their application as anodes in lithium-ion battery cells (LIBs). For PTPT@AC and POTPT@AC anodes, they showed a high capacity of 253.9 and 370.5 mA h g^−1^ at 100 mA g^−1^. Besides, the capacities of pure polymers were calculated to be 693.5 and 1276.5 mA h g^−1^ for PTPT and POTPT, respectively, at 100 mA g^−1^. Compared with PTPT, the introduction of the 3,4-ethylenedioxy unit into the side chain of the thiophene unit leads to substantially improved performance of POTPT due to the lowered LUMO energy levels of POTPT and the electron-rich feature of the EDOT unit. It is suggested that the structure-tuning strategy might be an effective method to prepare the new polymer-based anode for next generation LIBs with high performance and high safety.

## Abstract 2

The preparation procedure and cycling performance of the two polymer composites.

## Graphical abstract

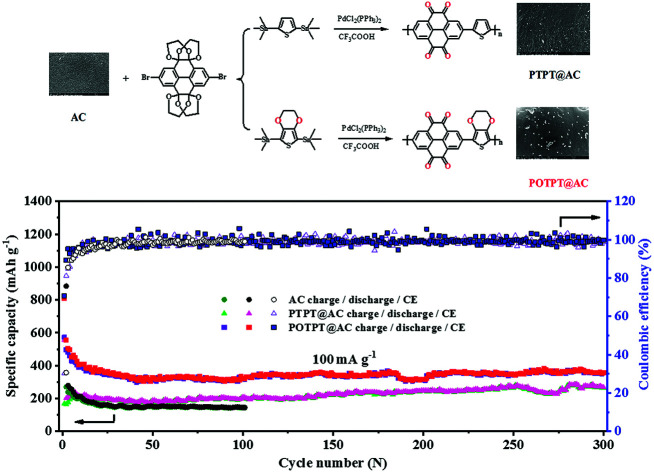

## 1. Introduction

Today, lithium-ion batteries (LIBs) play an irreplaceable role in many commercial explorations, including electric vehicles, electronic equipment, and energy storage.^1–3^ At the same time, research shows that commercially available electrode materials for LIBs are mainly inorganic substances, which are usually produced from non-renewable metallic minerals, and their manufacture usually brings huge energy costs and environmental pollution.^4–7^ Therefore, it is a pressing task to explore highly reliable electrode materials based on sustainable raw materials and green processes to satisfy the increasing requirements for green energy.^8–11^ Organic electrode materials can be improved in many aspects, including specific capacitance, stability and rate performance through more flexible means of structure tuning.^12–14^

Organic materials with lithium-loading functional units including benzene rings, C=N, and conjugated carbonyl compounds are considered as ideal organic electrode materials for LIBs;^15–21^ these kinds of materials include quinones,^22,23^ anhydrides, and ketones.^24,25^ The lithium storage course of these types of materials is fulfilled through the interaction between Li^+^ and their functional groups in a multi-step electron transfer process, which outputs a higher specific capacity. Particularly, much attention has been focused on pyrene-4,5,9,10-tetraone (PT), which has great capacities in potential due to the existence of two cyclic 1,2-diketone units in two six-membered rings, and can achieve the full utilization of all of its carbonyl groups as the PT unit was incorporated into the proper molecular system.^26^ Nokami *et al.*^17^ prepared a PT-bearing polymer polymethacrylate, which provided a capacity of 231 mA h g^−1^, high reliability, and high rate property. Xie *et al.*^26^ fabricated two polymer-based cathodes, including the homopolymers of PT and 2,7-ethynyl substituted PT, the latter polymer gave a higher capacity than the former polymer, which suggested that improvements in the conjugation length and planar properties of the latter polymer are helpful to improve the overall performance of the cathode materials. It is very helpful to increase the theoretical capacity of organic electrode materials by directly introducing PT into a long conjugation chain. The strategy not only provides an ideal carrier for PT but also regulates the redox behaviors of the functional groups during the Li^+^ intercalation/deintercalation process of conjugated polymers according to their energy levels and electronic structures.

Polymers of thiophene and their derivatives are another kind of important organic electrode material for LIBs. Poly(3,4-ethylenedioxythiophene) (PEDOT) has been adopted as a cathode material in LIBs, and a multi-step four-electron pathway was proposed for every EDOT unit in the charge/discharge process.^27^ In another report conducted by Zhang *et al.*,^28^ the poly(thiophene) (PT) was employed as the anode, and a multi-step four-electron pathway was also proposed for every thiophene unit.

It might be a feasible tactic to incorporate both the PT unit and the thiophene unit (or EDOT) into an extended conjugation system to achieve high-performance polymer-based electrode materials used in LIBs. Another important consideration for the construction of the copolymer between PT and thiophene (or EDOT) lies in the fact that the resulting copolymer may have the D–A type characteristic, since PT has an electron-deficient properties, and thiophene, especially EDOT has strong electron-donating property. The D–A type polymers are usually a kind of n-type doping polymers, having low band gaps, with the lowered LUMO and elevated HOMO energy levels, which endow the polymers with high electronic conductivity and low electrode potential. These properties make D–A type polymers very suitable as anode materials for LIBs. Meanwhile, the formation of composite materials between polymers and carbon nanotubes has been reported to enhance the conductivity of the organic electrode materials.^29^

In this study, we synthesized two D–A type copolymers consisting of alternating thiophene (or EDOT) and PT by the Stille coupling reaction, and then prepared their composites with activated carbon (AC) through the *in situ* polymerization method. The composites including PTPT@AC and POTPT@AC are studied in terms of their physical characteristics, charge–discharge performance, as well as lithium intercalation mechanisms as anode materials in LIBs. The resulting data suggest that both the composites could be adopted as anodes to render satisfactory capacities with high running stability. Notably, a conclusion has been reached that the side chain of the EDOT unit in POTPT could implement a stabilization effect on the inserted lithium ions, which is beneficial to increase the performance of the EDOT containing polymers. The report renders us with an approach to control the performance of organic polymer-based anodes with a slight change in the side chains of the electron-donating unit. To support the results of the study, changes in energy levels and electronic structures of the polymers after the Li^+^ intercalation was also calculated using density functional theory (DFT).

## 2. Experimental section

### 2.1. Materials, reagents and instrumental analysis

The materials, monomers and solvents involved in the synthesis, post-treatment process, and analysis experiments were obtained commercially. Together, detailed information on the instrumental analysis can be obtained in the ESI.† Besides, the detailed process of Natural Bond Orbital (NBO) analysis is also given in the ESI part.† The monomer of 2,7-dibromo-pyrene-4,5,9,10-tetraone (2BrPT) was first transformed into EPT to prevent the possible inactivation effect of the *ortho*-diketones on the catalyst during the Stille coupling reaction through chelating interactions, the detailed procedure is referred to in the ESI.†

### 2.2. Synthesis of materials

The synthesis of PTPT@AC composite materials is as follows. First, 438.44 mg (0.732 mmol) of EPT, 300 mg of 2SnTh (0.732 mmol), and 29.3 mg of PdCl_2_(PPh_3_)_2_ were added to a round bottom flask. Then, 1002.0 mg of AC for supercapacitors and 30.00 ml of toluene were added to the flask. The chemical mixture was placed in a water bath sonicator for 30 minutes to mix it more evenly. The reaction solution was refluxed at 110 °C for 48 hours under the protection of inert gas. For purification, the product was filtered, and the solid product was extracted with *n*-hexane, methanol, and acetone for 24 hours in a Soxhlet extractor and then the black composite material was obtained. The resultant product was dispersed in a mixture of TFA–H_2_O (9 : 1, 40 ml) in an airtight single-port flask, and fluxed under an argon atmosphere for 24 hours. Afterward, the mixture was cooled to room temperature, and the precipitate was obtained by filtration, washed thoroughly with excessive water to neutral pH, and then washed with methanol two times to give a black product. In the obtained PTPT@AC composite, the polymer PTPT accounts for 20% of the total mass, and polymer POTPT also accounts for 20% of the POTPT@AC composite. The same procedure was also suitable for the synthesis of POTPT@AC (See ESI†).

### 2.3. Electrochemical measurement

To prepare the working electrode, the given masses of PTPT@AC and POTPT@AC composites were thoroughly mixed with acetylene black and PVDF at a mass ratio of 70 : 20 : 10. NMP was added during the mixing process to obtain a uniform slurry, which was then ground for 2 hours using a colloid mill. The slurry was then coated on copper foil substrate (thickness: 100 μm), vacuum-dried at 60 °C for 24 hours, and then sliced to form a circular electrode with a diameter of 12 mm, which was used as the working electrode. The electrode was further vacuum-dried at 120 °C overnight. The final weight of the active material on each electrode was about 0.65–0.80 mg. The electrode sheets were then transferred to an argon-filled glove box. A lithium disk was used as the counter electrode to assemble the CR2032 coin battery. The supporting electrolyte was prepared by dissolving 1.0 M LiPF_6_ in a mixture of ethylene carbonate and dimethyl carbonate (*v* : *v* = 1 : 1). Galvanostatic charge–discharge (GCD) test was performed on the LANHE blue-cell battery test system in a voltage range of 0.005–3 V (unless otherwise stated, the potential used in this paper is opposite to that of Li^+^/Li). The capacity was calculated based on the total mass of the active material loaded on the working electrode. Cyclic voltammetry (CV) measurements were performed on an Autolab PGSTAT302N, scanning at various scan rates over a potential range of 0.005–3 V. Nyquist plots were recorded using Autolab PGSTAT302N at 100 kHz to 0.1 Hz. In order to make the electrode ready, the surface of the glassy electrode was polished with Al_2_O_3_ (0.3 μm) slurry, and ultrasonic cleaning was performed in ultra-pure water to achieve mirror polishing. Then the slurry of the POTPT material (or PTPT) was made, which consisted of 1.6 mg of POTPT (or PTPT), 1.6 mg of acetylene black, 0.32 mg of PVDF and 1 ml of NMP in a 1.5 ml centrifuge tube. The mixture was treated with ultrasound for 30 min until a well-dispersed ink was formed. Finally, 4 μl of the ink was dipped onto the electrode and then was dried at room temperature before testing. CV tests were performed with a conventional three-electrode system, with 0.2 mol l^−1^ TBAPF_6_/ACN solution, with a potential window between −2 V–2 V (*vs.* pseudo Ag wire as a reference electrode), and the sweep rate was 100 mV s^−1^.

## 3. Results and discussion

### 3.1. Morphology characterization

**Scheme 1:**
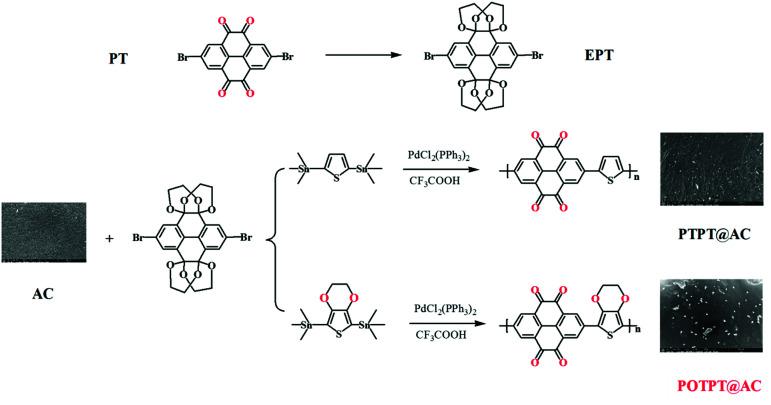
Synthesis routes of PTPT@AC and POTPT@AC.

**Fig. 1:**
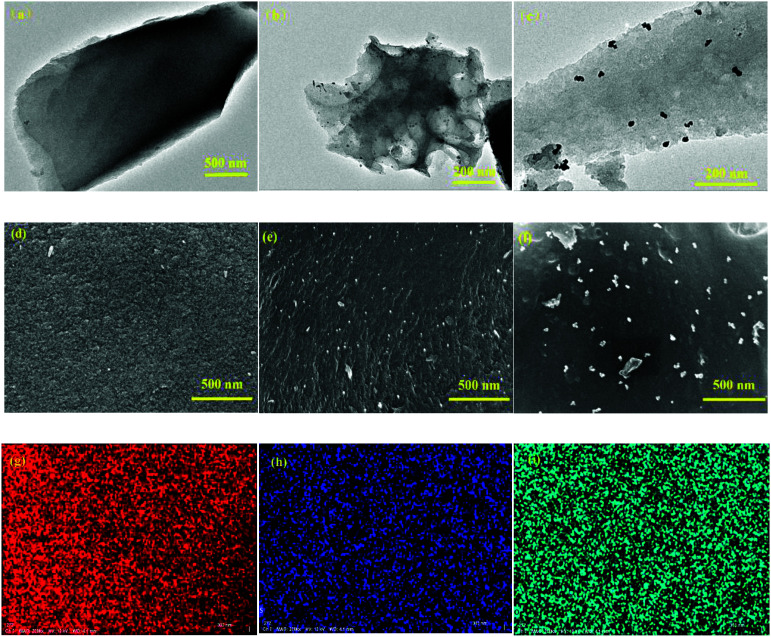
TEM images of the AC (a), the PTPT@AC (b), and the POTPT@AC (c), SEM images of AC (d), PTPT@AC (e) and POTPT@AC (f), the corresponding EDS spectrum of POTPT@AC and elemental mapping of (g) carbon, (h) sulfur, and (i) oxygen.

The two polymers with donor–acceptor (D–A) characteristics were prepared by the Pd catalyzed polymerization method, as shown in Scheme 1. To avoid the deactivation of the Pd catalyst by *ortho*-diketones, the PT monomer was modified by an etherification reaction with ethylene glycol solution by the catalysis of TsOH·H_2_O, and the modified monomer was designated to EPT. The corresponding polymers were synthesized by the Stille reaction between EPT and organotin compounds of thiophene or EDOT.^26^ The raw products were purified using the Soxhlet extraction to enhance the electrochemical stability of PTPT@AC or POTPT@AC composite materials. The TEM images of AC, PTPT@AC, and POTPT@AC are shown in Fig. 1a–c, respectively. Compared with AC, PTPT@AC, POTPT@AC showed an outer loose layer on the surface of the carbon powder, and the dotted black small particles might have been formed by the self-aggregation of polymers. Fig. 1d–f show SEM images of AC, PTPT@AC, and POTPT@AC, respectively. AC showed a rough and flat morphology (Fig. 1d), and the PTPT@AC composite presented a rough surface with wrinkles on it (Fig. 1e), suggesting a polymer layer was formed on the surface of the carbon powder. In comparison, the surface of POTPT@AC was more compact and dotted with white granular matter (Fig. 1f). Similarly, the SEM data suggest that the bright particles scattered on the surface of the composites may be polymer nanoparticles formed by the self-aggregation of polymer segments during the synthesis stage. The results suggested that the polymer can not only form a layer film on the surface of the carbon powder but also form nanoparticles through self-aggregation. Fig. 1g–i and S1a–c† are the EDS spectra of the corresponding composite materials of POTPT@AC and PTPT@AC. Three elements including C, O, and S contained in both polymers were evenly distributed in the composite material with an amorphous structure. Polymer@AC composites are formed by the coating of polymers on carbon particles during the polymerization process of monomers, and composites are relatively uniform, although there are scattered polymer nanoparticles distributed on the surface of the composite.

### 3.2. Structure confirmation by FT-IR, Raman spectrum, and XPS

**Fig. 2:**
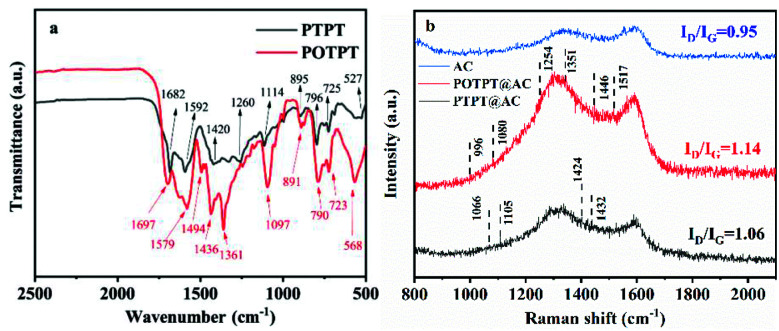
(a) FT-IR of PTPT and POTPT, (b) Raman spectrum of PTPT@AC, AC, and POTPT@AC.

The FT-IR tests were performed on these two polymers (without AC in the synthesis process) to confirm the compositions of the polymers. The test results are shown in Fig. 2a. According to infrared spectra of the two polymers, it is obvious that some of their absorption peaks are relatively close, because they use the same monomer, the EPT monomer in the Stille coupling polymerization. Taking POTPT as an example, the C=O bond is present in the PT monomer, its stretching vibration (SV) is confirmed by the peak at 1697 cm^−1^.^30^ The broad peaks at 1579 to 1436 cm^−1^ are caused by skeletal vibrations of PT or the EDOT ring.^29^ The peak at 1097 cm^−1^ was due to SV of the C–O–C bond present in the EDOT unit of the POTPT polymer.^31^ The absorption peaks at 891, 790, 723, and 568 cm^−1^ are referred to as the out-of-plane C–H bending vibration of the C–H bond on the skeleton of the POTPT polymer. The peak at 1361 cm^−1^ was due to the C–S SV of the thiophene (or EDOT) unit. For the polymer PTPT, the SV peak at 1682 cm^−1^ was attributed to the C=O bond in the PT unit, and the peak at 1592 cm^−1^ was due to the skeletal vibration of the C=C bond in the PT frame or the thiophene frame.^29^ The peaks at 895, 796, 725 and 527 cm^−1^ are the out-of-plane C–H bending vibration of the C–H bond on the backbone of the PTPT polymer. Further, the peak at 1114 cm^−1^ was due to the in-plane bending vibration of the C–H bond on thiophene or the PT ring.^32,33^ Raman spectroscopy was also employed for getting complementary information to the FT-IR data. As shown in Fig. 2b, Raman spectra of PTPT@AC, POTPT@AC composites, and AC show two distinct bands at about 1340 and 1580 cm^−1^, the former band is related to the disordered carbon (D band), the latter band is related to the graphitic carbon (G band). The peak intensity ratio of D-band/G-band (*I*_D_/*I*_G_) indicates the degree of the disordered carbon in the sample. After calculation, *I*_D_/*I*_G_ ratios of PTPT@AC, POTPT@AC, and AC are 1.06, 1.14, and 0.95, respectively. Compared with AC, PTPT@AC, POPTC@AC exhibit an increase in the intensity ratio of *I*_D_/*I*_G_, indicating higher disorder and defect structure of the composites.^29^ In the Raman spectrum of POTPT@AC, typical peaks at 1517, 1446, 1351, 1254, 1080, and 996 cm^−1^ are due to the asymmetric C_α_=C_β_ stretching, symmetric stretching of C_α_=C_β_, C_α_=C_β_ inter-ring stretching, C_α_–C_α_ inter-ring stretching, C–O–C deformation, and C–C anti-symmetrical stretching in the polymer skeleton of POTPT,^34^ respectively, which supports the presence of the EDOT unit in the composite. The Raman spectrum of PTPT@AC exhibits four peaks at 1066, 1105, 1424, and 1432 cm^−1^ which may be ascribed to C–H bending, C–C stretching (anti), the C=C stretching in the quinoid form, and the C=C stretching, respectively,^35^ which confirms the presence of thiophene unit in the composite. The Raman spectra of POTPT and PTPT polymers are also shown in Fig. S6a and b,† respectively. For POTPT (Fig. S6a†), the typical peaks at 1583, 1445/1430, 1327, 1219, 1079 and 890 cm^−1^ are in accordance with those of POTPT@AC (Fig. 2b). The same phenomenon was observed for the PTPT polymer (Fig. S6b†), the spectrum showed the dominant peaks at 1591, 1448, 1433, 1174, 1066 and 887 cm^−1^, which can be ascribed to the polythiophenes.

**Fig. 3:**
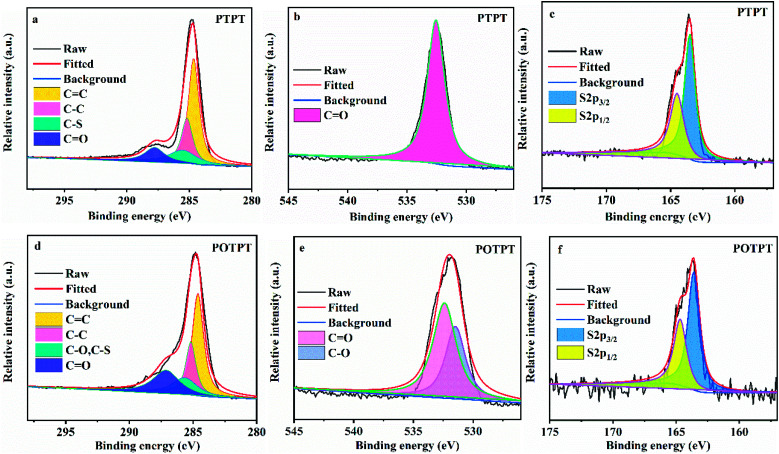
(a) C 1s, (b) O 1s, and (c) S 2p spectra of XPS results for PTPT. (d) C 1s, (e) O 1s, and (f) S 2p spectra of XPS results for POTPT.

To verify the feasibility of the synthesis method, the two polymers were also synthesized without the addition of carbon powder. XPS measurements were conducted to confirm the formation of polymers including PTPT (Fig. 3a–c) and POTPT (Fig. 3d–f). According to XPS peak positions of binding energies, it can be found that there are three characteristic elements of C, O, and S in both polymers. In the C1s spectrum, the peaks at 284.65, 285.18, 285.51, and 287.78 eV match the C̲=C, C̲–C, C̲–S, and C̲=O atoms,^16^ respectively in PTPT polymer (Fig. 3a). The peak with binding energy at 532.55 eV (Fig. 3b) corresponds to the C=O̲ atom on the PT unit. In addition, for the elemental S2p spectrum of the PTPT polymer (Fig. 3c), the binding energies at 163.50 and 164.50 eV match the 2p_3/2_ and 2p_1/2_, respectively, of the S atom (on the thiophene unit), respectively.^31^ Similarly, for the POTPT polymer (Fig. 3d) the peaks at 284.62, 285.17, 285.65, and 287.12 eV correspond to the C̲=C, C̲–C, C̲–S (or C̲–O), and C̲=O atoms, respectively. In Fig. 3e, the binding energies at 532.40 and 531.48 eV match those of C=O̲ and C–O̲ atoms, respectively, the former atom is on the PT unit and the latter atom is on the EDOT unit. For the POTPT polymer (Fig. 3f), the binding energies at 163.60 and 164.68 eV, match those for 2p_3/2_ and the 2p_1/2_ of the S element on the EDOT unit.^36^ All of the above results showed that PTPT and POTPT polymers can be successfully synthesized by the Stille coupling polymerization reaction. It is worth noting that XPS spectra of the corresponding elements of the PTPT@AC composite (Fig. S4a–c†) are almost the same as the XPS spectra of the PTPT polymer (Fig. 3a–c). This situation also occurs for POTPT@AC composites (Fig. S4d–f†) and POTPT polymer (Fig. 3d–f). The above discussions confirmed the formation of polymer@AC composites.

### 3.3. Physical characterization using XRD, TGA, and BET surface area

In Fig. S5a,† XRD patterns of PTPT@AC and POTPT@AC composite materials show similar diffraction characteristics, with a wide diffraction peak at about 2*θ* = 24°, corresponding to the (002) crystal surface of the graphite structure of the carbon material, which is a typical feature of amorphous carbon.^27^ In addition, the diffraction peak around 44° is very weak, corresponding to the (101) diffraction plane. The TGA curves of the two pure polymers are shown in Fig. S5b.† All polymers have high thermal stability at temperatures higher than 300 °C, enough for ensuring the safety of the long-term running of LIBs.^17^ The surface properties of the two composites and AC were also estimated from the nitrogen adsorption isotherms (Fig. S7a and b†), and the data indicate that the formation of composites between polymers and carbon powder can enhance the specific surface area of polymers and expose more active sites to the electrolyte solution, which can facilitate the transport and storage of lithium ions in the composites.^37^ The detailed information on the surface area and pore size distribution for all the samples can be found in ESI Note 1.†

### 3.4. The energy levels calculated from CV

**Fig. 4:**
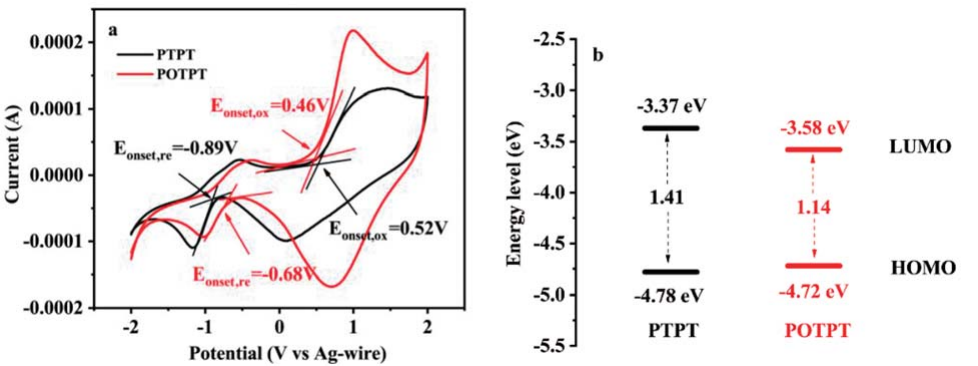
(a) CV curves in 0.2 mol l^−1^ TBAPF_6_/ACN electrolyte, the scan rate is 100 mV s^−1^. (b) Energy level diagram of PTPT and POTPT.

The electronic structure of polymers could play a significant role in their redox activities as well as in their storage capacities for lithium ions. To study these aspects, electrochemical band gaps and their energy levels were calculated using the CV method (Fig. 4a), from which, the proposed energy level diagrams of the two polymers are shown in Fig. 4b. The n-type doping phenomenon was observed for both polymers, which might be due to the strong electron affinity of the PT unit making the polymer accept electrons at the negative potentials. The detailed method for the calculation of the energy levels is provided in ESI Note 2.† The stronger electron-donating ability of the EDOT unit than that of the thiophene unit could be accounting for the lower bandgap of POTPT than that of PTPT (Fig. 4b). POTPT exhibits a lower LUMO energy level (−3.58 eV) than that of PTPT (−3.37 eV) (Fig. 4b), which indicates that POTPT has greater electron affinity and better oxidizability than that of PTPT. At the same time, POTPT shows a higher HOMO energy level (−4.72 eV) than that of PTPT (−4.78 eV), which suggests that POTPT is more conductive to lose its electrons at a more negative potential than that of PTPT. The low band gaps endowed the polymers with high electronic conductivity, and the lowered energy levels and the elevated HOMO energy levels make the polymers have low electrode potentials. The above properties of the two conjugated polymers make them particularly suitable as anode materials for LIBs.

### 3.5. The capacitance and EIS measurements of the composites

**Fig. 5:**
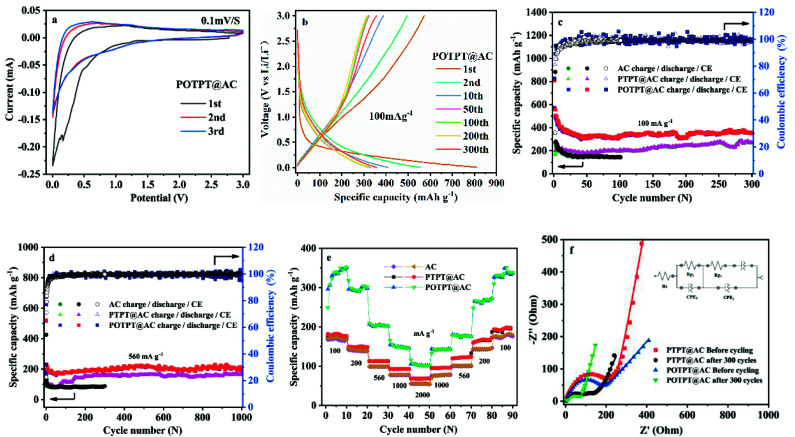
(a) Initial three cycles CV curves of POTPT@AC. (b) The galvanostatic charge–discharge (GCD) curves of POTPT@AC at 100 mA g^−1^. Running stability and coulombic efficiency (CE) of PTPT@AC and POTPT@AC at 100 mA g^−1^ (c), and at 560 mA g^−1^ (d). (e) Rate performance of PTPT@AC and POTPT@AC at different current densities. (f) Electrochemical impedance spectra (EIS) of PTPT@AC and POTPT@AC before cycling and after 300 cycles, which were tested at open circuit potential. The inset is the equivalent circuit model.

Fig. 5a and S8a† show the initial three CV curves of POTPT@AC and PTPT@AC anodes, at a scan rate of 0.1 mV s^−1^. The first CV cycle of the POTPT@AC electrode is different from the other two cycles (Fig. 5a), especially in the discharge region. For which, a weak peak was observed at 0.3 V, which is usually attributed to the formation of the SEI film, also the occurrence of some irreversible reactions.^38^ This phenomenon was also observed in other reports, which can lead to the high initial irreversible discharge capacity in the first discharge process.^39^ By contrast, the subsequent charging stage exhibits a much lower capacity, which leads to a low CE in the first discharge–charge process. From the second cycle, CV curves are principally coincident, which shows that the subsequent electrochemical process is reversible and stable.^26,38^ For POTPT@AC, the main redox reaction takes place between 0–1.5 V(*vs.* Li^+^/Li). Similarly, CV curves of PTPT@AC (Fig. S8a†) show a reduction peak at 1.0 V, while the asymmetric oxidation peak was at 1.25 V. The peak area in this interval is large, which may correspond to the reversible storage of lithium ions in composite materials. From Fig. 5b, the initial discharge and charge capacities of the POTPT@AC anode were measured as 808.8 and 572 mA h g^−1^ at 100 mA g^−1^, respectively. The calculated low CE of 70.72% is mainly ascribed to the decomposition of the electrolyte and the formation of the SEI film during the initial discharge,^16^ which is also the reason why charge and discharge curves in the first cycle are different from other cycles. For the first cycle, the open circuit potential was 2.54 V and the cell exhibits slopped GCD profiles without any apparent plateaus (Fig. 5b) and the capacities of POTPT@AC continuously decrease up to the 50th cycle (Fig. 5c). After 300 cycles, the POTPT@AC anode renders a reversible capacity of 370.5 mA h g^−1^ with a CE of 99% (Fig. 5c). It was observed that even at 560 mA g^−1^ (Fig. 5d), the POTPT@AC anode gave a reversible capacity of 214.5 mA h g^−1^, and it still maintained a stable CE of nearly 99% after 1000 cycles of the cycling process. In contrast, Fig. S8b† shows GCD profiles of the PTPT@AC anode, and the first discharge capacity was 556 mA h g^−1^, the first charge capacity was about 167.6 mA h g^−1^, leading to a low initial CE of about 30.14% (Fig. S8b†). The CE values could be quickly recovered to 81.11% at the second cycle and maintained at about 99% in the following cycles. At current densities of 100/560 mA g^−1^ (Fig. 5c and d), PTPT@AC and AC could deliver discharge capacities of 253.9/164.9 mA h g^−1^ and 144/84 mA h g^−1^, respectively. The results reveal that at the same current density composite material discharge showed much higher capacities than those of AC. The discharge/charge capacity of the PTPT@AC anode was somewhat lower than that of the POTPT@AC anode, which might indicate that POTPT has a higher capacity performance than that of the PTPT polymer. The practical capacity performances of the polymers are consistent with the prediction from the energy levels of polymers (Fig. 4b). Furthermore, the EDOT unit has a higher electron cloud density than that of the thiophene unit, this will stabilize the intercalated lithium ions during the discharge process, which would lead to the higher electrochemical performance of POTPT than that of PTPT. In addition, the good cycling performance of POTPT@AC should be attributed to the higher conjugation length and the resultant higher electronic conductivity of POTPT than that of PTPT. Rate performance is an important index of electrode materials, which is related to the realization of fast charging and discharging. The rate performances of PTPT@AC, POTPT@AC, and AC are shown in Fig. 5e, from which, the capacity retention ratios of PTPT@AC, POTPT@AC, and AC at 2000 mA g^−1^ were 37.36%, 30.37%, and 31.77%, respectively, of their average capacity at 100 mA g^−1^. When returned to 100 mA g^−1^, the capacities of the two composites could be well recovered, which demonstrates that the composites have excellent reversibility and structural stability. Based on the excellent characteristics of POTPT@AC, Table S2† lists the electrochemical performance comparison between polymer electrode materials for lithium-ion batteries. Fig. S1d and e† shows SEM images of PTPT@AC and POTPT@AC after the charge/discharge cycle.

EIS measurements were also conducted for PTPT@AC and POTPT@AC anodes between 100 kHz to 0.1 Hz with a disturbance voltage of 5 mV. The results are shown in Fig. 5f. The Nyquist plots of the two composites can be fitted and analyzed based on the equivalent circuit model (Fig. 5f). The curve was fitted according to the interpolation circuit diagram (Fig. 5f). *R*_p2_ is the diameter of a semicircle in the intermediate frequency region, which represents the charge transfer resistance between the electrode and the electrolyte surface, corresponding to the reaction kinetics of the electrode material,^29^ and the diagonal line indicates the Warburg impedance in the low-frequency region, that is, the capacitive reactance characteristic of the material, which is related to the Li-ion diffusion process. Generally speaking, the more perpendicular the straight line, the faster the ion migration rate is, which is advantageous for improving the electrochemical performance.^38^ As shown in Fig. 5f, the results showed that *R*_p2_ impedances of PTPT@AC and POTPT@AC, which were 240 Ω and 144 Ω, respectively, the EIS values were measured before GCD measurements. It is clear that POTPT@AC has a lower *R*_p2_ value than that of the PTPT@AC composite, showing a more rapid charge transfer process, which could lead to an excellent cycling performance of anodes. The charge transfer resistance of *R*_p2_ shows a slight decrease during the first 300 cycles of the charge/discharge process, and the final *R*_p2_ for PTPT@AC was 126 Ω, and the for POTPT@AC was 72.1 Ω, which might indicate that more ion-transport channels are formed in the running process due to the relaxation and swelling of the polymers.^40^

### 3.6. The lithium storage mechanism of the composite electrode

**Fig. 6:**
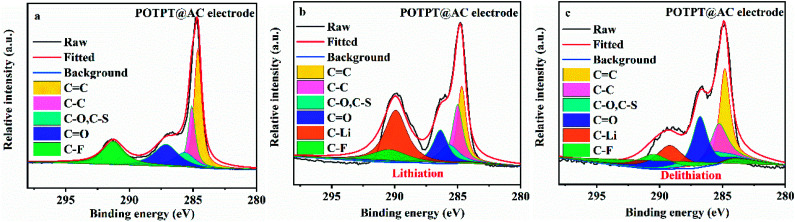
(a) C 1s spectra of XPS results for POTPT@AC electrode, (b) POTPT@AC electrode after lithiation, (c) POTPT@AC electrode after delithiation.

The XPS study was conducted to probe the Li storage mechanism of the composite electrodes. As shown in Fig. 6a, the POTPT@AC electrode has nearly identical XPS spectra with that of POTPT@AC, except that an additional peak at 291.26 eV (C̲–F in the binder) was observed. The Li storage mechanism by the composite could be discerned from the changes in C=C and C=O bonds during the lithiation/delithiation reactions, and the information could be obtained from the XPS measurement. After the lithiation (discharge) process (Fig. 6b), a new strong-weak peak at 289.92 eV indexed to C̲–Li emerges,^16^ which suggested the occurrence of the lithiation reaction with C̲=C in the conjugated structure of the composite. In addition, the peak intensity for C̲=C can be recovered after the charging process (Fig. 6c), indicating the reversible discharge process on C=C bonds. The above discussions suggested that lithium ions can be stored on C=C and C=O bonds. For the PTPT@AC electrode (Fig. S4g–i†), a similar phenomenon was also witnessed, suggesting the same lithium storage mechanism as discussed above.

### 3.7. The lithium-ion storage process of the composites

Since the pseudocapacitance occurs on or near the surface of the electrode material, it has a rapid ion diffusion performance. Therefore, it is very important to improve the contribution rate of pseudocapacitance for the exploration of high-quality electrode materials. The formation of composites between D–A type polymers and AC may substantially maintain the high specific surface area of the AC and also endow the composites with high conductivity, and these properties are beneficial for getting high pseudocapacitance of the composites. The CV curves under different sweep speeds are used to analyze the electrochemical reaction kinetics of electrode materials. The measured energy formula between the peak current intensity (*i*) and sweep speed (*v*) is as follows:

1 *i* = *av*^*b*^

2 log *i* = *b* log *v* + log *a*

**Fig. 7:**
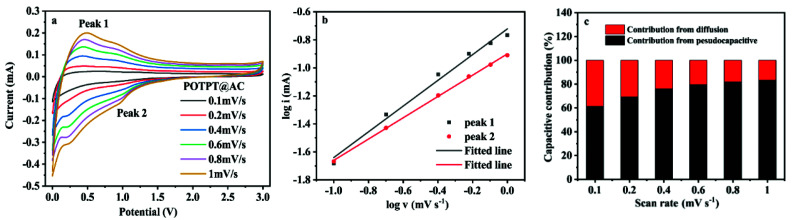
(a) CV curves of the POTPT@AC anode at different scan rates; (b) fitted lines log *v vs.* log *i* for the POTPT@AC anode; (c) capacitive contribution of POTPT@AC anode for LIBs.

The detailed analysis could be referred to the ESI Note 3,† and it is clear that the pseudocapacitance provides most of the capacitance contribution for POTPT@AC. The data shown in Fig. 7a–c support the same conclusion mentioned just above. For the two composites, the high pseudocapacitive contribution might be derived from the large specific surface area of the composites. In this case, the lithium ions are easy to be stored in active sites and the surface of materials, which is beneficial to improve the capacity and the running stability of the electrode materials.^36^

### 3.8. The structure–activity relationship of the polymers

Since AC was added during the material synthesis process, it is necessary to consider the capacity contribution of AC. Therefore, the capacities of the pure polymers can be calculated using the equation (a) shown in ESI Note 4,† in which, the contribution of AC was deduced. Cycling stabilities and CE of PTPT and POTPT at 100 mA g^−1^ and 560 mA g^−1^ are shown in Fig. S8c and d.† Deducing the contribution of AC from composites, PTPT and POTPT delivered discharge capacities of 693.5 mA h g^−1^ and 1276.5 mA h g^−1^ at 100 mA g^−1^, respectively (Fig. S8c†).

The minimum repeating unit of POTPT includes a PT unit and an EDOT unit. Similarly, the minimum repeating unit of PTPT includes a PT unit and a thiophene unit. According to a previous study,^16,18,41–43^ every C=O group can store one Li-ion, and every C=C group can store two Li-ions, every benzene ring can react with six Li-ions. As a result, the theoretical Li^+^ storage number for each repeating unit of the polymers are 20 for PTPT and POTPT (Fig. S10†); based on this, the theoretical capacity of PTPT and POTPT is 1558 mA h g^−1^ and 1333 mA h g^−1^, respectively. Each repeating unit of POTPT can accept 19.2 Li ions on average, which is very close to its theoretical storage number of 20. However, each repeating unit of PTPT can only accept 8.9 Li ions on an average, which is quite lower than its theoretical number of 20. The obtained capacities of polymers are often lower than their theoretical values since the active sites of the polymer do not fully participate in the Li-ion storage process due to the aggregation between the molecular chains, and are not accessible to the supporting electrolyte, and as a result, practical capacities are depressed.^44^

The higher lithium-ion storage performance of POTPT than that of PTPT might be largely due to the higher electronic conductivity of POTPT derived from its lower bandgap than that of PTPT (Fig. S11b†), which might enable fast π-electron migration along with the framework of the conjugated polymer. Besides, the higher the electron-donating ability of the EDOT unit along the polymer backbone can stabilize the lithium ions more effectively than that of the thiophene unit. The intercalation of lithium-ion and electrons in the polymer will change the energy level of the polymer, which will increase the HOMO value and decrease the LUMO value at the same time (Fig. S11a†), resulting in the decrease of the bandgap and the improvement of conductivity. For POTPT, its EDOT unit has a stronger electron-donating ability, and after the lithium-ion is introduced, the charge can be delocalized in the whole polymer backbone, and be promptly stabilized, and the LUMO energy level of the polymer could be controlled at a relatively stable level, which is conducive to the storage of subsequent lithium ions, thus being consistent with the higher reversible capacity of POTPT@AC than that of PTPT@AC. Besides, the bandgap values of both polymers in this study are much smaller than the values of most of the organic electrodes described in the previous literature.^26^ Therefore, the composites reported in this study are very suitable as anode materials for LIBs, which showed promising specific capacities and rate performance.

## 4. Conclusions

Two novel polymers consisting of alternating thiophene (EDOT) and PT units, were designed and synthesized, the polymers are designated as PTPT (thiophene containing) and POTPT (EDOT containing). The D–A properties of polymers endowed them low band gaps, which was characterized by lowered LUMO and elevated HOMO energy levels. In this case, the two polymers have low redox potentials at the same time, which are suitable as anode materials for LIBs. As anode materials in LIBs, POTPT@AC gave a capacity of 370.5 mA h g^−1^, and PTPT@AC gave 253.9 mAg g^−1^ at 100 mA g^−1^. The capacities of the pure polymers were also calculated, which were 693.5 and 1276.5 mA h g^−1^ (current density 100 mA g^−1^), respectively, for PTPT and POTPT, with the capacity of POTPT much higher than that of PTPT. The lower bandgap and the stronger electron-donating ability of the EDOT unit for POTPT polymer might be accounting for its higher electrochemical performance than that of PTPT. Overall, the construction of D–A type conjugated polymers with tunable structures using cross-coupling polymerization could provide new insights to develop new polymer-based anodes with high energy density and high stability.

## Data availability statement

All related data can be available upon request.

## Conflicts of interest

There are no conflicts to declare.

## Supplementary Material

RA-011-D1RA00794G-s001
