# Supplementary material for: The synthesis of alternating donor–acceptor polymers based on pyrene-4,5,9,10-tetraone and thiophene derivatives, their composites with carbon, and their lithium storage performances as anode materials
Source: RSC Adv. 2021 Apr 22;11(25):15044–53. doi: 10.1039/d1ra00794g (PMC8698373; doi:10.1039/d1ra00794g)
Supplement: RA-011-D1RA00794G-s001 [file RA-011-D1RA00794G-s001.pdf]

Electronic Supplementary Material (ESI) for RSC Advances.
This journal is © The Royal Society of Chemistry 2021

Supporting Information

The synthesis of the alternating donor-acceptor polymers based on pyrene-4,5,9,10-tetraone and thiophene derivatives, their composites with carbon and the lithium storage performances as anode materials

Xin Guo[a], Qing Yuan[a], Chunxia Li[a], Hongmei Du[a], Jinsheng Zhao[a, b]*, Lixia Liu, Yunwu Li[a,b]*, Yu Xie[c,]* Vijay Vaidya[d]

[a]Shandong Key laboratory of Chemical Energy Storage and Novel Cell Technology, Liaocheng University, Liaocheng, 252059, P.R. China

[b]College of Chemistry and Chemical Engineering, Liaocheng University, 252059, P.R. China.

[c] College of Environment and Chemical Engineering, Nanchang Hangkong University, Nanchang 330063, PR China

[d]Fu Technology, Co. Ltd.Tianjin, 851000, P.R. China

*Corresponding Author: j.s.zhao@163.com (J.S. Zhao); liyunwu@lcu.edu.cn(Y.W.Li); xieyu_121@163.com(Y. Xie)

Materials and reagents

The materials, monomers and solvents involved in the synthesis, post treatment process and analysis experiments are obtained from the commercial approach. 2,5-bis(trimethylstannyl) thiophene (2SnTh) were purchased from J &K scientific. 5,7-Bis(trimethylstannyl)-2,3-dihydrothieno [3,4-b][1,4] dioxine (2SnEDOT) is purchased from SunaTech Inc. 2,7-dibromo-pyrene-4,5,9,10-tetraone (2BrPT) and the catalyst $PdCl_2(PPh_3)_2$ are obtained from Zhengzhou Alfa Chemical Co., Ltd. Tetrabutylammonium hexafluorophosphate ($TBAPF_6$, 98%), acetonitrile (ACN) and N-

methyl-2-pyrrolidinone (NMP, 99.9%) were purchased from Aladdin Co., Ltd. Ethanol, acetone, toluene are bought from YanTai Far East Fine Chemical Co., Ltd. And the activated carbon (AC) for supercapacitors was purchased from XFNANO. Acetylene black, trifluroacetic acid (TFA), polyvinylidene fluoride (PVDF) and the $LiPF_6$ electrolyte were purchased from Liyuan Battery Sales Department, Yingze District, Taiyuan City.

The detailed information on instrumental analysis

The transmission electron microscopy (TEM) was taken to fulfil the structural characterization using JEM-2100. The particle morphologies of the as-prepared powder are also examined by a Hitachi Su-70 scanning electron microscopy (SEM, Hitachi Inc., Tokyo, Japan).The phase composition of each product was characterized by using powder XRD in the 2θ range from 5 to 80˚ by using a Rigaku D/max 2500 X-ray diffractometer, with Cu-Ka radiation, and a step scan mode was adopted with a scanning step of 0.02°. The data of Raman spectroscopy are obtained using a Horiba Scientific Raman Spectrometer with a 785 nm Ar ion CW laser source. X-ray photoelectron spectroscopy (XPS) was conducted with ESCALAB 250Xi spectrometer to determine the surface composition and the valence state of the elements within the as-prepared powder. The porosity was determined by nitrogen adsorption and desorption at 77 K using an ASAP 2460-3 (Micromeritics) volumetric adsorption analyzer. The thermogravimetric analyses of the samples were conducted on a Netzsch STA449C TG/DSC thermal analyzer under nitrogen atmosphere between 20°C and 800°C. Fourier transform infrared (FT-IR) spectra were performed by a Nicolet 6700 FTIR spectrometer. The cyclic voltammetry (CV) measurements were performed on an Autolab PGSTAT302N.

The detailed information on Natural Bond Orbital (NBO) Analysis

The Natural Bond orbital (NBO) analysis, which can be obtained from the density matrix of the system, was introduced to calculate the components of the atomic orbitals. In this work, the calculation was based on the HF/6-31G level. [1,2] Geometry optimizations are executed using the HF density functional implemented with Gaussian 09 without symmetry restriction. The 6 -31G (d, p) basis sets are adopted for H, Li, C, S and O atoms. The measurement of the LUMO of the polymers includes four steps: first, the contribution of each atom to the LUMO energy is calculated by the NBO analysis, Second, both the Li^+ and e^- are inserted to the atom which has the largest contribution to the LUMO energy level. Then, the molecule is recalculated by Gauss 09 for the new molecular structure with the lowest energy can be obtained. Finally, the LUMO of the new molecular structure are calculated by Gauss 09. The next all LUMO was determined following the same procedures mentioned above until the number of inserted Li^+ equals to the value measured by experiments.

Synthesis of EPT

In a round bottom flask, 756 mg (1.8 mmol) of PT, 684 mg (3.6 mmol) of $TsOH{\cdot}H_2O$, 18 mL of ethylene glycol and 18 mL of toluene were added in order. The resultant mixture was heated in a oil bath and be maintained at 140 °C under the protection of argon gas for 36 hours. The reaction mixture was then cooled down to room temperature, the precipitant was recovered by filtration, washed with excess water and small amount of MeOH, dried and the final product was obtained as a light yellow solid (650 mg, yield:

61%). Figure S2 and Figure S3 show the ^{1}H NMR and ^{13}C NMR of EPT . ^{1}H NMR ($CDCl_3$, 500 MHz): δ 7.893 (s, 4H), 4.207 (s, 8H), 3.667 (s, 8H); ^{13}C NMR ($CDCl_3$, 500 MHz): δ 134.978, 130.335, 127.432, 123.813, 91.943; HRMS: Calculated for $C_{24}H_{20}Br_2O_8+H^+$: 598.9562; Found: 598.9551 ($M+\ H^+$); Elemental Analysis for $C_{24}H_{20}Br_2O_8$: Calculated: C, 48.35%; H, 3.38%. Found: C, 48.11%; H, 1.91%.

The synthetic procedure for POTPT@AC

The synthesis of POTPT@AC composite materials was as that of the PTPT@AC composite. For this purpose, 383.93 mg (0.641 mmol) of EPT and 300 mg of 2SnEDOT (0.641 mmol) and 35 mg of $PdCl_2(PPh_3)_2$ were added to a round bottom flask. Then, 1027 mg of the activated carbon (AC) and 30 mL of toluene were added to the flask. By following the same preparation method as that of PTPT@AC and then the black POTPT@AC composite material was successfully obtained. Also, the polymer POTPT accounts for 20% of the POTPT@AC composite material.

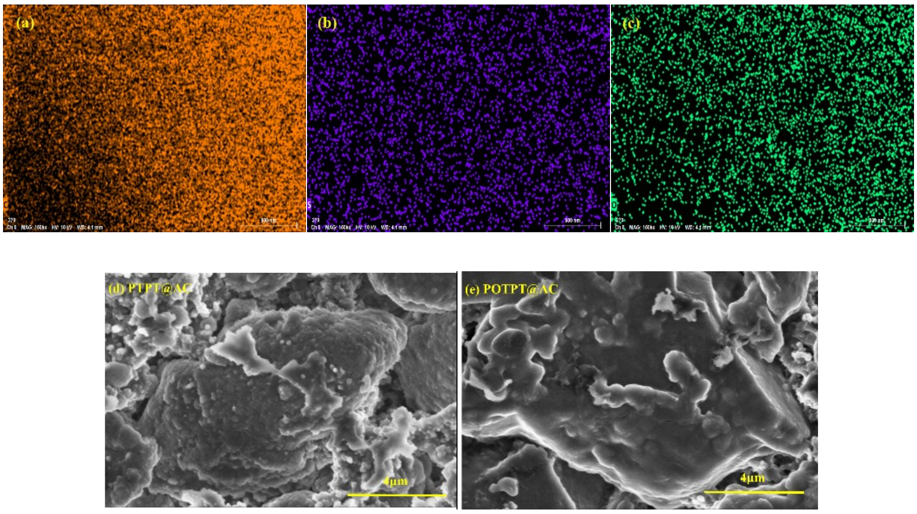

Figure S1. The corresponding EDS spectrum of PTPT@AC and elemental mapping of (a) carbon, (b) sulfur, and (c) oxygen. SEM images of PTPT@AC (d) and POTPT@AC

(e) after charge/discharge cycle.

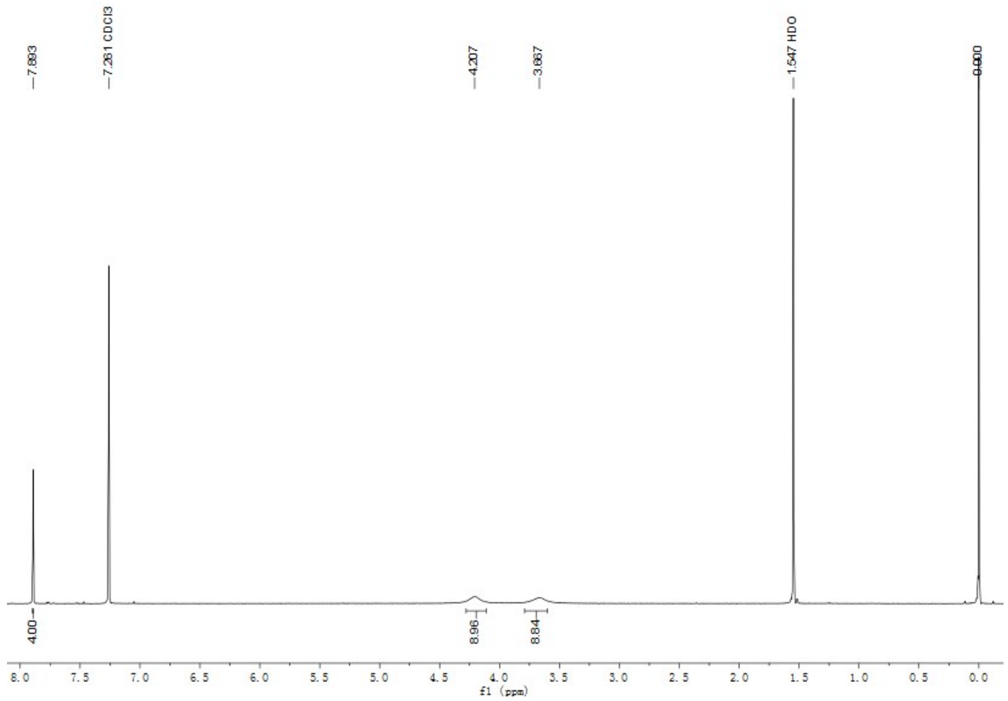

Figure S2 ^{1}H NMR of EPT

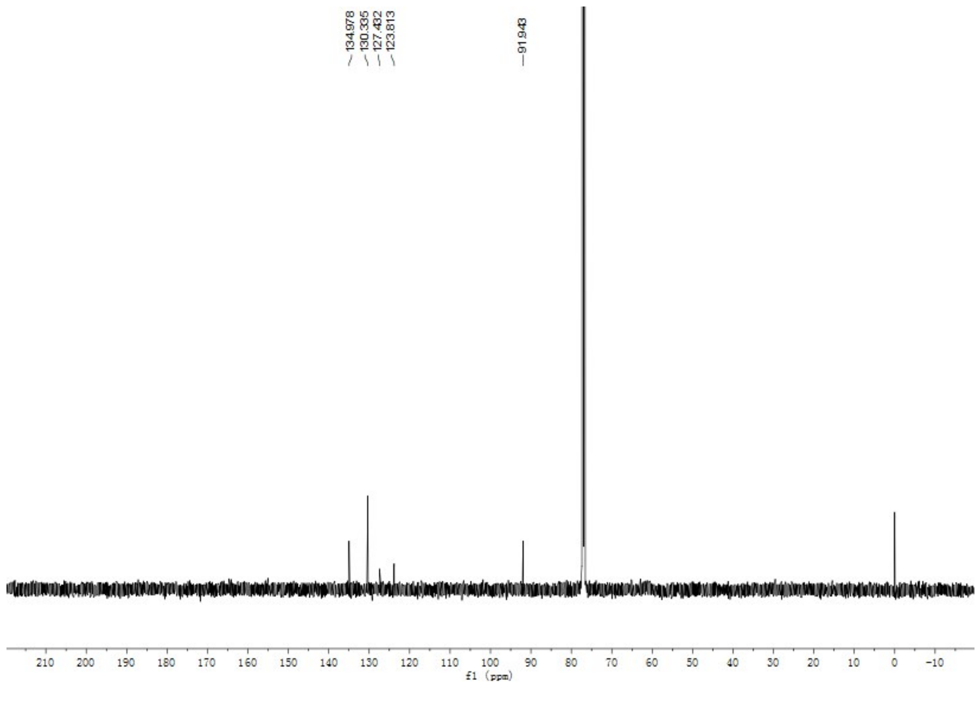

Figure S3 ^{13}C NMR of EPT

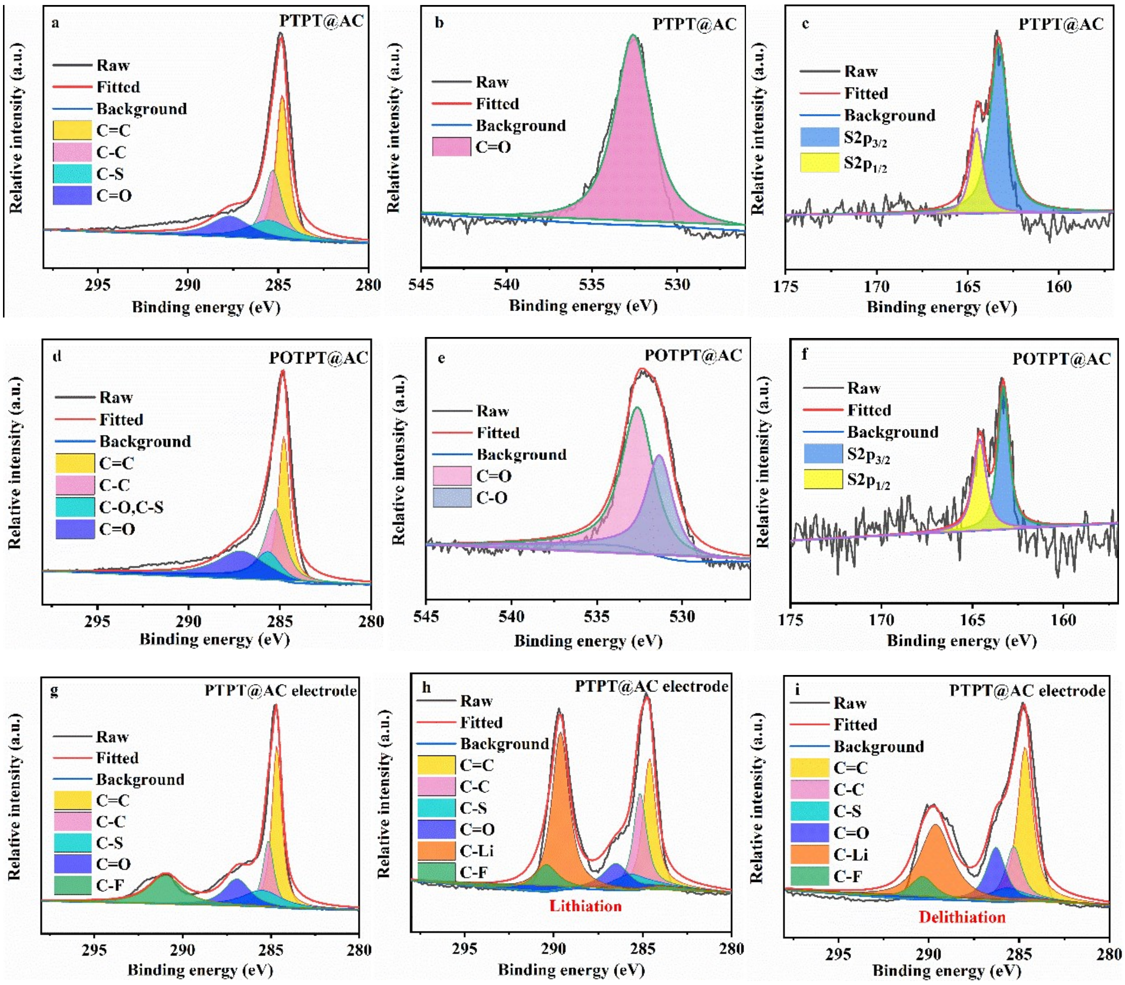

Figure S4. (a) C 1s, (b) O 1s, (c) S 2p spectra of XPS results for PTPT@AC. (d) C 1s, (e) O 1s, (f) S 2p spectra of XPS results for POTPT@AC. (g) C 1s spectra of XPS results for PTPT@AC electrode, (h) PTPT@AC electrode after lithiation , (i) PTPT@AC electrode after delithiation.

Supplementary Note 1

Thermogravimetric analysis showed that the pure PTPT or POTPT (the AC is not involved in the synthesis process) have high thermal stability (Figure S5b). From the TGA curves, their initial decomposition temperatures (T_d, at which the weight loss is 5%) can be readily got, which were 318.3 °C for PTPT and 308.2 °C for POTPT. [3] All polymers

have high thermal stability at temperatures higher than 300 °C, which is an important feature from the perspective of the safety of the lithium-ion battery. [4]

Figure S7a and Figure S7b show the nitrogen adsorption isotherms and pore size distribution of PTPT@AC and POTPT@AC at 77K. The adsorption isotherm curves of both composites present the type I isotherm behavior, showing the existence of the micropores. [5,6] At relatively low pressure, capillary condensation and langmuir single-layer reversible adsorption process occur, and the adsorption capacities of the samples are controlled by the pore volume. The turning points of the platforms of the isotherms are corresponding to the case, where the pores of the adsorbents are completely filled with condensed liquid. The specific surface areas, average pore sizes, and total pore volumes are calculated using the Brunauer-Emmett-Teller (BET) and Barrett-Joyner-Halenda (BJH) methods, which are listed in Table S1. Both of the corresponding values for the composites are considerably lower than that of the AC, which might be caused by the coating of the polymers on the surface of the AC and even blocked some of the channels on the surface of the composites. The formation of the composites between the polymers and the carbon powder can enhance the specific surface area of the polymers and expose more active sites to the electrolyte solution, which can facilitate the transport and storage of lithium ions in the composites. [6] The above-mentioned micromorphological characteristics of the composites could enhance the capacities of the composites by enlarging the area of the of the solid electrolyte interface (SEI) membrane formed during the initial discharge.[7-11]

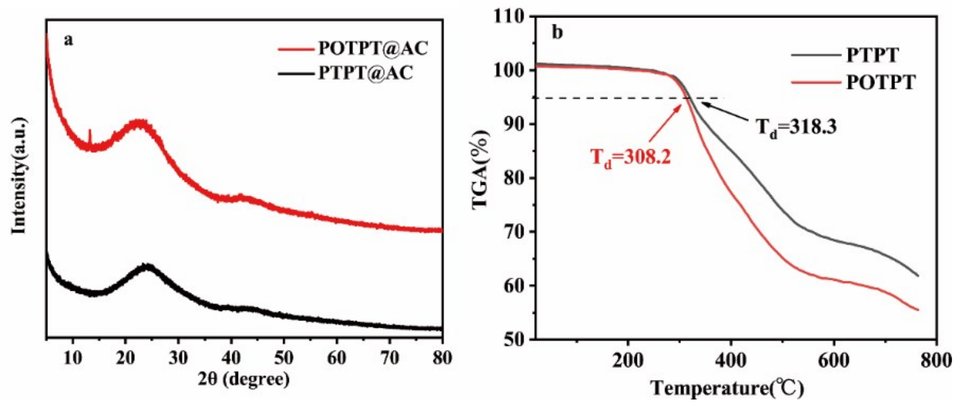

Figure S5. (a) XRD of PTPT@AC and POTPT@AC. (b) TGA curves of PTPT and POTPT under N_2 atmosphere.

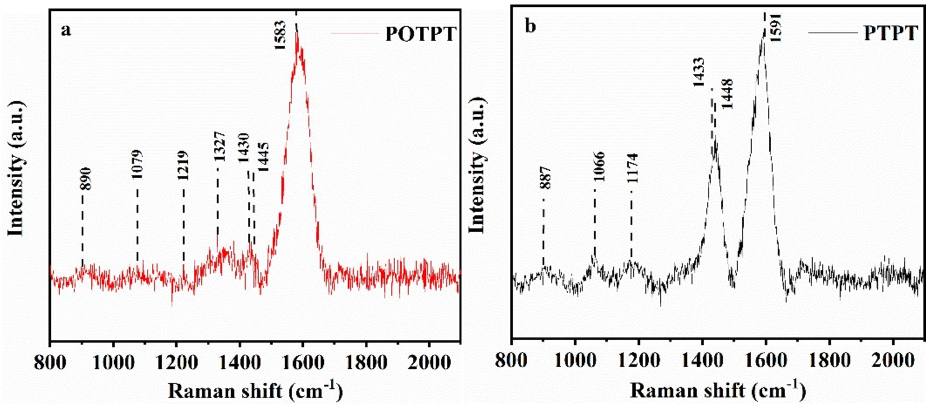

Figure S6. Raman spectrum of POTPT(a) and PTPT(b).

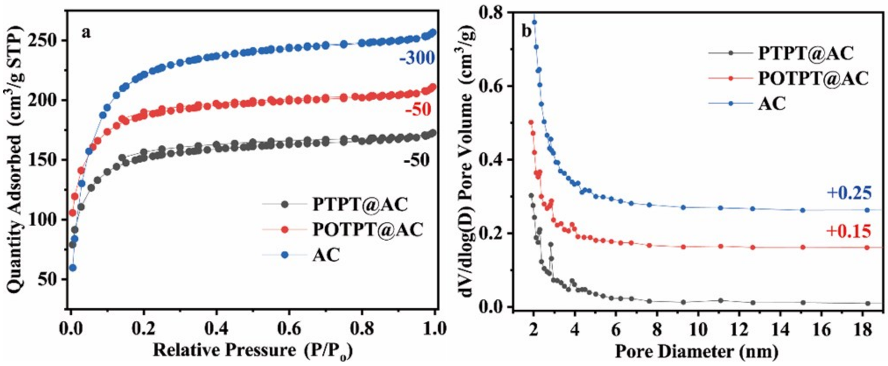

Figure S7. (a) Nitrogen adsorption-desorption isotherms, and (b) pore size distribution curves of PTPT@AC, POTPT@AC and AC.

Table S1. The specific parameters on the micro-morphology of AC and two composites

Samples	S_{BET} (m^2/g)	Mean pore diameter(nm)	Total pore volume(cm^3/g)
AC	1581.3	2.178	0.861
PTPT@AC	619.4	2.225	0.345
POTPT@AC	726.7	2.223	0.404

Supplementary Note 2

Interestingly, as shown in Figure 4a, both of the two polymers showed apparent n-type doping property, as evidenced by the appearance of the redox peaks at the negative potentials. For polymer POTPT, it has an $E_{onset,ox}$ value of 0.46 V, and an $E_{onset,re}$ value of -0.68 V, and consequently has a low bandgap of 1.14 eV. For polymer PTPT, it has an $E_{onset,ox}$ value of 0.52 V, and an $E_{onset,re}$ value of -0.89 V, and the has the bandgap value of 1.41 eV. The valence band (VB) and conduction band (CB) positions of the polymers could be calculated from the onset potentials of them using equations (1) and (2), and then the electronic band alignments can be drawn and shown in Figure 4b. The number 0.54 in the formula is a correction parameter, which was used to calibrate the Ag wire electrode to the half wave potential of F_c/F_c^+.

$$E_{HOMO}=-[4.8+(E_{onset,ox}-0.54)]eV \qquad (1)$$

$$E_{LUMO} = -[4.8+(E_{onset,re}-0.54)]eV \quad (2)$$

$$E_g = E_{LUMO} - E_{HOMO} \quad (3)$$

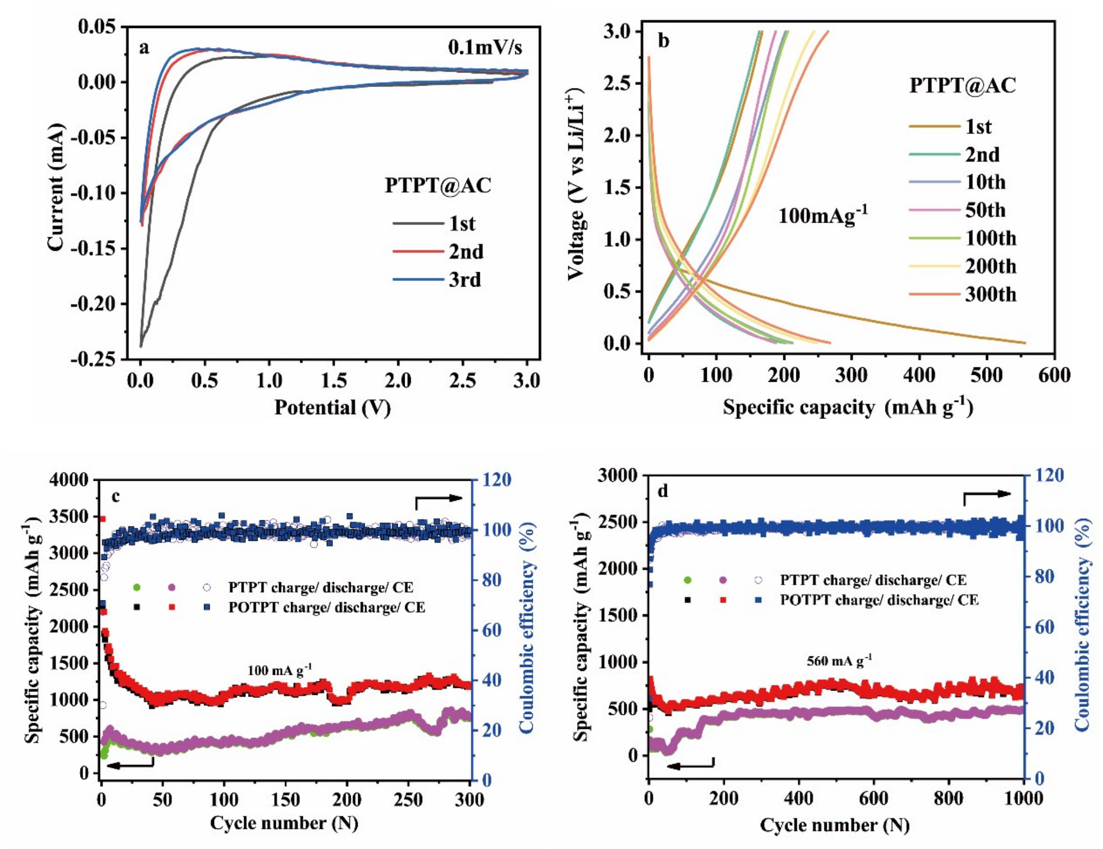

Figure S8. (a) Initial three CV curves of PTPT@AC (b)The GCD curves of PTPT@AC at 100mA g^{-1}. Cycling stability and Coulombic efficiency of PTPT and POTPT (c) at 100 mA g^{-1} and (d) 560 mA g^{-1}.

Supplementary Note 3

It is very important to understand the source of the capacities of electrode materials and then study their lithium ion storage mechanisms for revealing the structure-activity relationships of them. Some physical properties of electrode materials, such as the particle size, porosity, specific surface area, conductivity, phase structure, etc., had profound

effects on the specific capacities of these materials. In addition, some deintercalation and redox reactions related to the molecular structure of electrode materials are also important factors affecting their practical performance.[12,13]

Since the pseudocapacitance occurs on or near the surface of the electrode material, it has a rapid ion diffusion performance. However, the traditional ion diffusion occurs within the electrode material, which is controlled by the diffusion rate in the solid phase, usually the reaction rate is slow. Therefore, it is very important to improve the contribution rate of pseudocapacitance for the exploration of high quality electrode materials. The formation of the composites between the D-A type polymers and the AC may substantially remain the high specific surface area of the AC and also endow the composites with high conductivity, and these properties are beneficial for getting high pseudocapacitance of the composites.

Traditionally, the CV curves under different sweep speeds are used to analyze the electrochemical reaction kinetics of electrode materials. The measured energy formula between peak current intensity (i) and sweep speed (v) is as follows:

$$i = av^b \qquad (4)$$

$$\log i = b\log v + \log a \qquad (5)$$

Where a and b are constants to be determined, which can be obtained by the linear fitting of the log i-log v curve, the slope is the value of b. As the value of b approaches to 0.5, it indicates that the electrochemical process is governed by the internal solid-phase diffusion process. However, as the value of b approaches to 1, it indicates that the electrochemical reaction is mainly governed by the pseudocapacitive process.[14]

Furthermore, the whole capacity contribution can be divided into two parts, and accordingly the current value (i) from CV can be divided into the current from pseudocapacitance (k_1v) and the current from internal solid-phase diffusion ($k_2v^{1/2}$) control processes, which can be expressed by the following formula:

$$i = k_1v + k_2v^{1/2} \qquad (6)$$

Based on this, we have measured the CVs of PTPT@AC and POTPT@AC anodes at different scanning speeds. The results are shown in Figure S9a and Figure 7a. The peak current intensities (i) are fitted with the values of the scanning rates (v), and the relationship between them follows the above equation. The oxidation peak 1 was selected for data fitting, and the b values obtained were 0.89 and 0.92 for PTPT@AC (Figure S9b) and POTPT@AC (Figure 7b), respectively. The data from reduction peak 2 was also use for linear fitting, and the obtained b values were 0.77 and 0.76 for PTPT@AC(Figure S9b) and POTPT@AC (Figure 7b), respectively, which were close to 1. Therefore, it as believed that the capacity contribution is controlled by the pseudocapacitive behavior,[14] and indicated that the formation of the composites was an feasible way to enhance the lithium storage capacity of the polymers.

When the scanning speed is 0.1, 0.2, 0.4, 0.6, 0.8 and 1.0 mV/s, the pseudocapacitance contribution percentages of PTPT@AC anode (Figure S9c) is 63.3%, 70.9%, 77.5%, 80.9%, 83.0% and 84.5%, respectively. It is shown that the pseudocapacitive behavior of lithium ion storage has a great contribution to the overall capacity. In Figure 7c, the results showed that the contribution rate of the pseudocapacitance of POTPT@AC is 61.3% (0.1 mV/s), 69.2 % (0.2 mV/s), 76.0% (0.4 mV/s),79.5% (0.6 mV/s), 81.8% (0.8 mV/s) and

83.4% (1 mV/s), respectively, which indicated that the pseudocapacitance accounts for a large proportion (all over 50%) in the whole capacity contribution at different sweep speeds, and the high pseudocapacitive contribution might derived from the large specific surface area of the composites. In this case, the lithium ions are easy to be stored in the active sites and the surface of the materials, which is beneficial to improve the capacity and the running stability of the electrode materials.[5,11]

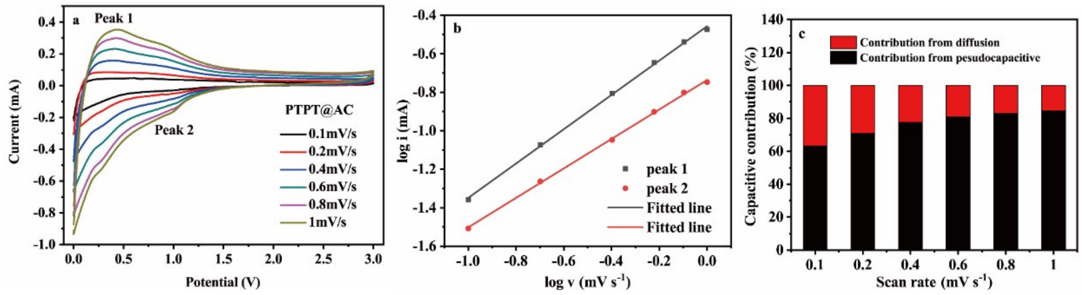

Figure S9. (a) CV curves of the PTPT@AC anode at different scan rates; (b) fitted lines log v vs. Log i for the PTPT@AC anode; (c) capacitive contribution of PTPT@AC anode for LIBs.

Supplementary Note 4

$$SC_{polymer} = \frac{SC_{total} \times M_{total} - SC_{carbon} \times M_{carbon}}{M_{polymer}} \quad \text{(a)}$$

Where, $SC_{polymer}$ is the specific capability of polymer (mAh g^{-1}), SC_{total} is the specific capability considering both polymer and active carbon (mAh g^{-1}), SC_{carbon} is the specific capability of active carbon (mAh g^{-1}), M_{carbon}, $M_{polymer}$ and M_{total} is the mass of the active carbon. polymer and polymer@AC composite (g), respectively.

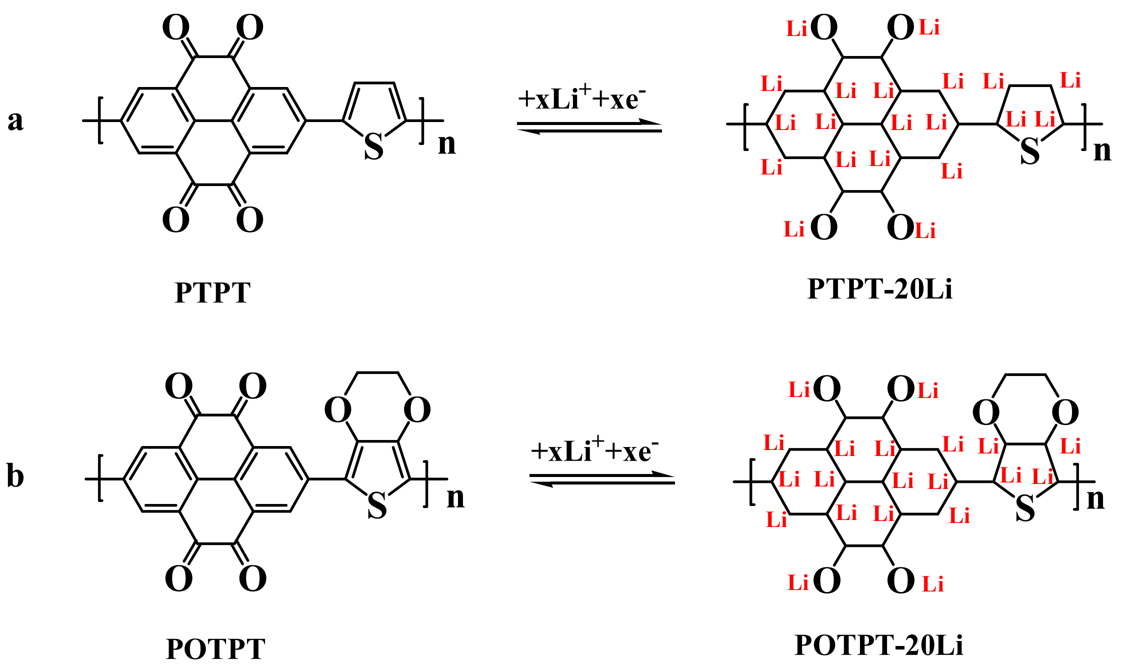

Figure S10. Schematic diagram of the proposed electrochemical reaction, single molecule structure and active site for storage of Li in (a) PTPT and (b)POTPT

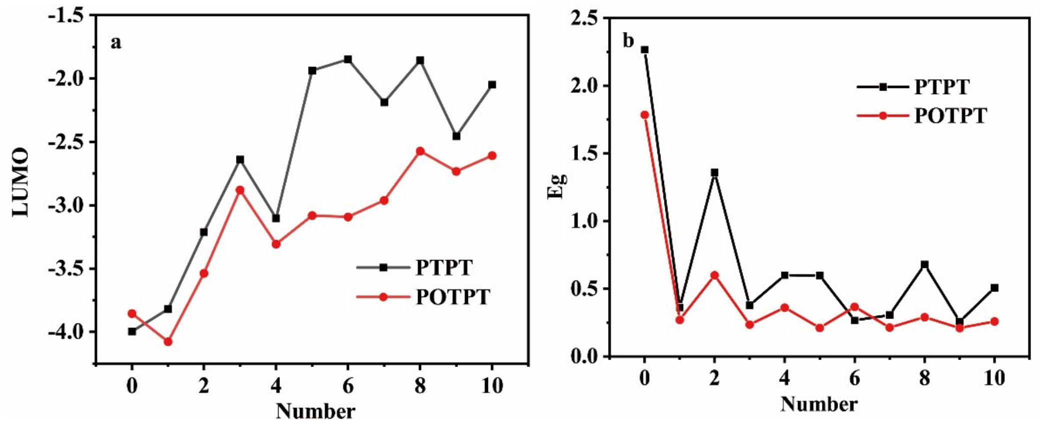

Figure S11. (a) The LUMO energy level of PTPT and POTPT (b) the Eg energy level of PTPT and POTPT.

Table S2. The electrochemical property comparison among the polymer electrode materials for lithium-ion batteries.

Sample	Practical capacity ($mAh\ g^{-1}$)	Cycle numbers/current density	Test condition	Reference
PTPT@AC	253.9 $mAh\ g^{-1}$	300 cycles / 100 $mA\ g^{-1}$	0.005-3V	this work
POTPT@AC	370.5 $mAh\ g^{-1}$	300 cycles / 100 $mA\ g^{-1}$	0.005-3V	this work
PPTC	87.2 $mAh\ g^{-1}$	300 cycles / 500 $mA\ g^{-1}$	1.5-3.5V	[15]
PPTO	161 $mAh\ g^{-1}$	100 cycles / 20 $mA\ g^{-1}$	1.5-3.5V	[16]
PEPTO	155 $mAh\ g^{-1}$	100 cycles / 20 $mA\ g^{-1}$	1.5-3.5V	[16]
PPYT	193 $mAh\ g^{-1}$	500 cycles / 1 C	1.5-3.5V	[4]
PI-1	129 $mAh\ g^{-1}$	100 cycles / 0.2 C	1.5-3.5V	[17]
PI-2	171 $mAh\ g^{-1}$	100 cycles / 0.2 C	1.5-3.5V	[17]
PI-3	156 $mAh\ g^{-1}$	100 cycles / 0.2 C	1.5-3.5V	[17]
PI-4	173 $mAh\ g^{-1}$	100 cycles / 0.2 C	1.5-3.5V	[17]
PI-5	183 $mAh\ g^{-1}$	100 cycles / 0.2 C	1.5-3.5V	[17]
PQI-1	135.6 $mAh\ g^{-1}$	200 cycles / 100	1.5-3.5V	[18]

		mA g^{-1}		

References

1. J. D. Dill and J. A. Pople , *J. Chem. Phys.*, 1975, **62** , 2921 - 2923.

2. W. J. Hehre, R. Ditchfield and J. A. Pople , *J. Chem. Phys.*, 1972, **56**, 2257 - 2261.

3. T. C. Chu, X. P. Ju, X. Han, H. M. Du, Y. Zhang, J. S. Zhao and J. H. Zhang , *Org. Electron.*, 2019, **73**, 43 - 54.

4. T. Nokami, T. Matsuo, Y. Inatomi, N. Hojo, T. Tsukagoshi, H. Yoshizawa, A. Shimizu, H. Kuramoto, K. Komae, H. Tsuyama and J. Yoshida , *J. Am. Chem. Soc.*, 2012, **134**, 19694 - 19700.

5. P. X. Wang, Y. Zhang, Y. Y. Yin, L. S. Fan, N. Q. Zhang and K. N. Sun , *ACS Appl. Mater. Interfaces*, 2018, **10**, 11708 - 11714.

6. D. Mukherjee, G. Gowda Y. K, H. Makri Nimbegondi Kotresh and S. Sampath , *ACS Appl. Mater. Interfaces*, 2017, **9**, 19446 - 19454.

7. D. Yan, C.Y. Yu, X. J. Zhang, W. Qin, T. Lu, B.W. Hu, H. L. Li and L. K. Pan , *Electrochim. Acta*, 2016, **191**, 385 - 391.

8. D. Su, K. Kretschmer and G. Wang , *Adv. Energy Mater.*, 2016, **6** , 1501785.

9. B.-S. Lee, S.-B. Son, K.-M. Park, W.-R. Yu, K.-H. Oh and S.-H. Lee , *J. Power Sources*, 2012, **199**, 53 - 60.

10. X. Q. Xie, S. J. Wang, K. Kretschmer and G. X. Wang , *J. Colloid Interface Sci.*, 2017, **499**, 17 - 32.

11. B. Ni, Y. Q. Li, T. Q. Chen, T. Lu and L. K. Pan , *J. Colloid Interface Sci.*, 2019, **542** 213 - 221.

12. X. J. Zhang, W. Ou-Yang, G. Zhu, T. Lu and L. K. Pan , *Carbon*, 2019, **143**, 116 - 124.

13. X. J. Zhang, G. Zhu, M. Wang, J. B. Li, T. Lu and L. K. Pan , *Carbon*, 2017, **116**, 686 - 694.

14. S. Li, J. X. Qiu, C. Lai, M. Ling, H. J. Zhao and S. Q. Zhang, *Nano Energy*, 2015, **12**, 224 - 230.

15. Q. Li, D. N. Li, H. D. Wang, H.-G. Wang, Y. H. Li, Z. J. Si and Q. Duan , *ACS Appl. Mater. Interfaces*, 2019, **11**, 28801 - 28808.

16. J. Xie, W. Q. Chen, G. K. Long, W. B. Gao, Z. C. J. Xu, M. Liu and Q. C. Zhang , *J. Mater. Chem. A*, 2018, **6**, 12985 - 12991.

17. Z. P. Song, H. Zhan and Y. H. Zhou , *Angew. Chem.-Int. Edit.*, 2010, **49**, 8444 - 8448.

18. B. B. Tian, G. H. Ning, W. Tang, C. X. Peng, D. Y. Yu, Z. X. Chen, Y. L. Xiao, C. L. Su and K. P. Loh, *Mater. Horiz.*, 2016, **3**, 429 - 433.
